# Daily versus alternate-day oral iron in women with iron deficiency anemia: impact of dosing schedule and adherence on hemoglobin recovery

**DOI:** 10.3389/fnut.2026.1893130

**Published:** 2026-07-27

**Authors:** Christian Omar Ramos Peñafiel, Emmanuel Martínez Moreno, Adolfo Martínez Tovar, Ana Vero Sosa, Carlos Martínez Murillo, Adán Germán Gallardo Rodríguez

**Affiliations:** 1 Hematology Department, Hospital General de México “Dr. Eduardo Liceaga”, Mexico City, Mexico; 2 Molecular Biology Department, Hospital General de México “Dr. Eduardo Liceaga”, Mexico City, Mexico; 3 Social Service in Health Research Program, Research in Medicine, Universidad Autónoma Metropolitana (UAM), Mexico City, Mexico; 4 School of Sport Sciences, Universidad Anáhuac México, Mexico City, Mexico

**Keywords:** dosing schedule, iron-deficiency anemia, oral iron supplementation, treatment adherence, women of reproductive age

## Abstract

**Introduction:**

Iron deficiency anemia (IDA) is common among women of reproductive age and is frequently associated with abnormal uterine bleeding (AUB). Although oral iron is first-line therapy, the optimal dosing schedule remains uncertain. This study compared daily and alternate-day oral iron in real-world practice.

**Materials and methods:**

We conducted a prospective observational cohort study of women aged 18–70 years with confirmed IDA treated with oral ferrous sulfate at a tertiary referral center between January 2023 and June 2024. Patients received oral iron every 24 h or every 48 h according to physician prescription. AUB etiology was classified using the PALM–COEIN system. Outcomes included hemoglobin change, World Health Organization anemia severity, hemoglobin normalization (≥12 g/dL), adherence, adverse-event-related discontinuation, and intravenous iron escalation.

**Results:**

A total of 171 women were included. Leiomyomas were the most frequent PALM–COEIN category (106 patients, 62.0%). Mean baseline hemoglobin was 9.02 ± 1.45 g/dL. At 6 weeks, mean hemoglobin was 10.91 ± 1.76 g/dL in the 24-h group and 11.26 ± 1.86 g/dL in the 48-h group (*p* = 0.255). At 3 months, final hemoglobin was available in 147 patients; mean values were 12.79 ± 1.68 g/dL and 12.68 ± 1.62 g/dL, respectively (*p* = 0.692). Hemoglobin normalization occurred in 75.2 and 71.7% of patients, respectively (*p* = 0.687). Adherence was high, and discontinuation due to adverse events was uncommon. Intravenous iron escalation was numerically more frequent with alternate-day therapy (26.8% vs. 13.9%; *p* = 0.056), although escalation decisions were based on clinical judgment without standardized criteria.

**Conclusion:**

Both regimens were associated with hematologic improvement. However, because allocation was non-randomized, baseline differences were present, and intravenous iron escalation was not based on standardized criteria, equivalence cannot be inferred. Oral iron dosing should be individualized.

## 1 Introduction

Iron deficiency anemia (IDA) remains the most common nutritional deficiency worldwide and disproportionately affects women of reproductive age (1). Globally, an estimated 30–40% of women of reproductive age have anemia, and iron deficiency accounts for nearly half of these cases (2). Heavy menstrual bleeding and abnormal uterine bleeding are among the leading causes of IDA in this population, often resulting in chronic blood loss, iron depletion, and reduced functional capacity (3). Oral iron supplementation is the first-line therapy for uncomplicated IDA due to its low cost, wide availability, and favorable safety profile. However, treatment success is frequently limited by poor gastrointestinal tolerance and suboptimal adherence, ultimately affecting hemoglobin recovery.

Despite widespread use, current clinical guidelines provide limited direction on optimal dosing frequency for oral iron supplementation in IDA (4). Traditional recommendations favor daily oral iron dosing, typically based on empirical practice rather than physiological evidence (3). Recent research, however, suggests that less frequent dosing regimens, such as alternate-day administration, might offer comparable efficacy with improved tolerability by mitigating hepcidin-mediated absorption inhibition and reducing gastrointestinal side effects (5). Despite these potential benefits, the optimal oral iron supplementation strategy for women with IDA, particularly concerning its impact on hemoglobin recovery and the role of adherence, remains unclear (6).

Real-world data in women with IDA remain scarce, and adherence has not been systematically evaluated as a modifying factor of treatment response. Most available trials involve small samples, selected populations, or short follow-up periods, and few studies specifically examine the clinical impact of adherence on hemoglobin normalization when comparing daily versus alternate-day dosing (7–9). Understanding whether the dosing schedule or adherence plays a greater role in determining treatment success is clinically relevant for optimizing therapeutic recommendations in primary care, hematology, and gynecology.

Therefore, this study aimed to compare hemoglobin recovery in women with iron deficiency anemia treated with either daily (every 24 h) or alternate-day (every 48 h) oral iron supplementation over 6 weeks, and to assess the role of treatment adherence in determining hematologic outcomes.

## 2 Materials and methods

### 2.1 Study design and setting

This was a prospective observational cohort study conducted at the Department of Hematology of Hospital General de México “Dr. Eduardo Liceaga” from January 2023 to June 2024. Women with newly diagnosed iron deficiency anemia (IDA) receiving outpatient oral iron supplementation were consecutively recruited during routine clinical evaluation. The study adhered to the principles of the Declaration of Helsinki and was approved by the local Research and Ethics Committee (DI/19/103/03/039). All participants provided written informed consent before enrollment.

### 2.2 Participants

Women aged 18 to 70 years with a confirmed diagnosis of iron deficiency anemia were eligible. Iron deficiency anemia was defined as hemoglobin <12.0 g/dL in combination with biochemical evidence of iron deficiency, including serum ferritin <30 ng/mL and/or transferrin saturation (TSAT) < 20%, according to routine clinical practice.

Serum ferritin was considered the primary diagnostic marker when available. In patients without available ferritin measurements or without clearly reduced ferritin values, iron deficiency was confirmed using complementary iron studies, including TSAT <20%, serum iron, total iron-binding capacity, and soluble transferrin receptor when available, together with the clinical context and complete blood count findings.

Exclusion criteria were: (1) pregnancy or breastfeeding at enrollment; (2) active infection, inflammatory disease, or chronic kidney disease; (3) history of gastrointestinal disorders affecting iron absorption, such as celiac disease or inflammatory bowel disease; (4) intravenous iron therapy or red blood cell transfusion within the previous 3 months; (5) current use of medications known to significantly modify iron absorption, such as proton pump inhibitors or tetracyclines; and (6) inability to attend scheduled follow-up visits or anticipated inability to complete adherence assessment.

Patients with abnormal uterine bleeding were included if they met diagnostic criteria for iron deficiency anemia. Patients receiving anticoagulant or antiplatelet therapy were not excluded unless they presented with active bleeding unrelated to abnormal uterine bleeding or met any of the predefined exclusion criteria.

Etiological classification of abnormal uterine bleeding was performed according to the PALM–COEIN system. Structural causes were categorized as polyp (P), adenomyosis (A), leiomyoma (L), and malignancy or hyperplasia (M), whereas nonstructural causes were categorized as coagulopathy (C), ovulatory dysfunction (O), endometrial disorder (E), iatrogenic cause (I), and not otherwise classified (N). Classification was based on information documented in the medical record, including clinical history, laboratory findings, imaging studies, histopathologic reports, and gynecologic assessment when available.

### 2.3 Intervention and follow-up

Participants received oral ferrous sulfate providing 115 mg of elemental iron, combined with folic acid 1 mg, vitamin B12 25 μg, vitamin C 600 mg, and vitamin E 25.83 mg. Oral iron was prescribed either as daily dosing every 24 h or alternate-day dosing every 48 h, according to physician prescription in routine clinical practice.

Treatment allocation was non-randomized and was determined by the treating physician during routine clinical practice. The choice of daily or alternate-day dosing was based on clinical judgment and patient-level considerations, including baseline anemia severity, prior intolerance to oral iron, gastrointestinal symptoms, perceived risk of poor adherence, ongoing blood loss, and patient preference. Therefore, treatment assignment was not controlled by the study protocol and may have introduced selection bias and baseline differences between groups.

All participants were instructed to take oral iron on an empty stomach with water and to avoid calcium-containing foods or beverages within 2 h of intake. Follow-up assessments were performed at baseline, 6 weeks, and 3 months when available. Hemoglobin values, anemia severity, adverse events, treatment discontinuation, adherence, and treatment escalation were recorded during follow-up.

### 2.4 Primary outcomes

The primary hematologic outcome was hemoglobin response during follow-up. As a continuous outcome, hemoglobin response was assessed by hemoglobin concentration at follow-up and by change in hemoglobin concentration from baseline. As a categorical outcome, hemoglobin normalization was defined as hemoglobin ≥12.0 g/dL, according to the World Health Organization threshold for anemia in non-pregnant women.

Hemoglobin values were also categorized according to World Health Organization anemia severity criteria: severe anemia was defined as hemoglobin <8.0 g/dL, moderate anemia as 8.0–10.9 g/dL, mild anemia as 11.0–11.9 g/dL, and normal hemoglobin as ≥12.0 g/dL. This classification was used to describe changes in anemia severity over time and to compare the distribution of hematologic response between dosing schedules.

Secondary outcomes included treatment adherence, treatment discontinuation due to adverse events, and escalation to intravenous iron therapy.

### 2.5 Adherence assessment

Treatment adherence was assessed using a structured patient-reported questionnaire and pill-count verification when available. Adherence was operationalized as high adherence when ≥80% of prescribed doses were taken and low adherence when <80% of prescribed doses were taken. The 80% threshold was selected because it is widely used in medication adherence research as a pragmatic cut-off to distinguish adherent from non-adherent patients (10).

Given the fixed elemental iron dose of 115 mg per administration, the alternate-day regimen resulted in approximately half the cumulative oral iron exposure over the 6-week period compared with the daily regimen. This difference reflected routine clinical practice and was not modified for the purposes of the study.

### 2.6 Intravenous iron escalation

Escalation to intravenous iron therapy was recorded as a secondary clinical outcome. The decision to initiate intravenous iron was made by the treating hematologist during routine follow-up and was not determined by random assignment. Criteria considered for escalation included inadequate hemoglobin response after oral therapy, persistent symptomatic anemia, ongoing abnormal uterine bleeding, intolerance or discontinuation of oral iron, need for faster hematologic correction, or physician concern regarding insufficient iron repletion.

Intravenous iron was generally considered after clinical and laboratory reassessment during follow-up, particularly after the 6-week evaluation or before the 3-month assessment when clinically indicated. No standardized institutional protocol mandated escalation to intravenous iron; therefore, this outcome reflects real-world treatment intensification in routine clinical practice.

### 2.7 Statistical analysis

Quantitative variables were described as means and standard deviations or medians and interquartile ranges, according to their distribution. Categorical variables were reported as frequencies and percentages.

Comparisons between the daily and alternate-day dosing groups were performed using Student’s t-test or the Mann–Whitney *U* test for continuous variables, as appropriate. Categorical variables were compared using the chi-square test or Fisher’s exact test, as appropriate.

To search for baseline differences between dosing groups, exploratory adjusted analyses were performed. Final hemoglobin concentration was evaluated using multivariable linear regression. Hemoglobin normalization and intravenous iron escalation were evaluated using multivariable logistic regression. Covariates were selected *a priori* based on clinical relevance and included dosing schedule, baseline hemoglobin concentration, age, and comorbidity status. Quantitative bleeding severity was not systematically recorded and therefore could not be included as an adjustment variable.

Changes in hemoglobin concentration from baseline to follow-up were evaluated using paired statistical tests. Hemoglobin normalization was analyzed as a binary outcome using the World Health Organization threshold of ≥12.0 g/dL. Associations between treatment schedule, adherence, comorbidity burden, endocrinologic disorders, and hemoglobin normalization were estimated using odds ratios with 95% confidence intervals. A *p*-value <0.05 was considered statistically significant. Statistical analyses were performed using SPSS version 25.

All eligible patients included in the final cleaned database were analyzed for baseline characteristics. Follow-up hemoglobin outcomes were analyzed using available cases only. Patients without final hemoglobin measurements at 3 months were excluded from analyses of final hemoglobin concentration and hemoglobin normalization because no follow-up hemoglobin values were available for these individuals. No imputation was performed for missing hemoglobin, ferritin, adherence, or follow-up data.

To assess the potential impact of missing follow-up data, a sensitivity analysis was performed comparing patients with and without available final hemoglobin measurements at 3 months using baseline variables available for all participants. Comparisons included age, dosing schedule, baseline hemoglobin concentration, baseline anemia severity, comorbidity status, baseline ferritin availability, and baseline ferritin values when available. Treatment discontinuation due to adverse events and escalation to intravenous iron were analyzed as observed clinical outcomes according to the information available in the medical record.

No formal *a priori* sample size calculation was performed because this was an exploratory real-world observational cohort based on consecutive eligible patients treated during the study period. Therefore, the study was not powered to establish equivalence, non-inferiority, or to exclude clinically meaningful differences between dosing schedules.

## 3 Results

A total of 171 women with iron deficiency anemia were included in the final analysis. The mean age was 39.26 ± 9.21 years, with an age range of 18 to 70 years; 121 patients (70.8%) were older than 35 years. Final hemoglobin measurements at the 3-month follow-up were available for 147 patients (86.0%), who were included in the available-case analysis for final hematologic outcomes.

Etiological classification according to the PALM–COEIN system showed a predominance of structural causes (PALM), mainly uterine leiomyomas (L), identified in 106 patients (62.0%), followed by adenomyosis (A) in 14 patients (8.2%) and endometrial polyps (P) in 10 patients (5.8%). No cases of endometrial malignancy or hyperplasia (M) were identified. Among nonstructural causes (COEIN), ovulatory dysfunction (O) was present in 27 patients (15.8%), followed by endometrial disorders (E) in 10 patients (5.8%) and coagulopathies (C) in 7 patients (4.1%). Additionally, 3 patients (1.8%) presented iron deficiency anemia secondary to early pregnancy loss occurring before study enrollment. These individuals were not pregnant at inclusion and fulfilled all eligibility criteria. Remaining cases were categorized as not yet classified (N) according to the PALM–COEIN system.

Overall, 54 patients (31.6%) had at least one documented comorbidity, whereas 117 patients (68.4%) had no relevant medical history. Among patients with comorbidities, endocrine and metabolic disorders were the most frequent group (Table 1).

**Table 1:** Concomitant medical conditions among patients with iron deficiency anemia.

Medical condition	*n* (%)
Endocrine and metabolic disorders	
Hypothyroidism	16 (29.6)
Hyperthyroidism	6 (11.1)
Diabetes mellitus or related metabolic conditions	6 (11.1)
Cardiovascular and thrombotic conditions	
Hypertension or previous thrombotic events	10 (18.5)
Autoimmune and hematologic disorders	
Rheumatoid arthritis	2 (3.7)
Immune thrombocytopenia	3 (5.6)
Antiphospholipid syndrome	1 (1.8)
Systemic lupus erythematosus	1 (1.8)
Autoimmune hepatitis	1 (1.8)
Sickle cell disease	1 (1.8)
Acute promyelocytic leukemia in remission	1 (1.8)
Gastrointestinal and surgical conditions	
Previous gastric bypass or chronic gastrointestinal disorders	6 (11.1)
Other less frequent conditions	
HIV infection	1 (1.8)
Breast cancer	1 (1.8)
Ovarian cancer	1 (1.8)
Asthma	1 (1.8)
Fibromyalgia	1 (1.8)
Psychomotor delay	1 (1.8)

Data are presented as *n* (%). Comorbidity status percentages were calculated using the final analytic cohort as the denominator (*n* = 171). Percentages for specific comorbidities were calculated among patients with at least one documented comorbidity (*n* = 54). Categories are not mutually exclusive because some patients had more than one concomitant medical condition.

### 3.1 Baseline hemoglobin levels and anemia characteristics at diagnosis

Overall, patients presented with moderate anemia at diagnosis, with a mean baseline hemoglobin concentration of 9.02 ± 1.45 g/dL. According to the World Health Organization classification, most patients had moderate anemia, followed by severe anemia and mild anemia.

Red blood cell indices showed a predominantly microcytic pattern, and red cell distribution width was elevated in most patients. Baseline ferritin was available in 103 patients (60.2%), with most available values falling below 30 ng/mL. In patients without available ferritin measurements or without clearly reduced ferritin levels, iron deficiency was confirmed using complementary iron studies and routine clinical assessment. Baseline hematologic and iron parameters are summarized in Table 2.

**Table 2:** Baseline hematologic and iron parameters.

Variable	Overall cohort *n* = 171
Hemoglobin at diagnosis	
Hemoglobin, g/dL	9.02 ± 1.45
Median hemoglobin, g/dL	8.9
Hemoglobin range, g/dL	5.5–12.0
WHO anemia severity	
Severe anemia, <8.0 g/dL	44 (25.7)
Moderate anemia, 8.0–10.9 g/dL	112 (65.5)
Mild anemia, 11.0–12.0 g/dL	15 (8.8)
Red blood cell indices	
Mean corpuscular volume, fL	69.53 ± 9.67
Median mean corpuscular volume, fL	67.8
Mean corpuscular volume range, fL	50.0–110.0
Microcytosis, MCV < 80 fL	145 (84.8)
Macrocytosis, MCV > 100 fL	1 (0.6)
Red cell distribution width, %	20.77 ± 5.57
Median red cell distribution width, %	19.4
Red cell distribution width range, %	12.9–47.8
Ferritin at diagnosis	
Ferritin available	103 (60.2)
Ferritin, ng/mL	8.5 (3.47–24.05)
Ferritin range, ng/mL	1.0–236.86
Ferritin <15 ng/mL	65/103 (63.1)
Ferritin 15–30 ng/mL	15/103 (14.6)
Ferritin ≥30 ng/mL	23/103 (22.3)

WHO, World Health Organization; MCV, mean corpuscular volume. Data are presented as mean ± standard deviation, median interquartile range, range, or *n* (%), as appropriate. Percentages for anemia severity, red blood cell indices, and ferritin availability were calculated using the full analytic cohort as the denominator (*n* = 171). Percentages for ferritin categories were calculated among patients with available baseline ferritin measurements (*n* = 103).

### 3.2 Comparison of oral iron dosing schedules (24-h dosing vs. 48-h dosing)

At baseline, patients receiving daily dosing had a lower mean hemoglobin concentration than those receiving alternate-day dosing. The distribution of baseline anemia severity showed a borderline between-group difference, with a greater proportion of mild anemia in the alternate-day group.

At the 6-week evaluation, hemoglobin increased in both groups. Mean hemoglobin was 10.91 ± 1.76 g/dL in the daily dosing group and 11.26 ± 1.86 g/dL in the alternate-day group, without a statistically significant between-group difference. The distribution of anemia severity at 6 weeks was not significantly different between groups.

**Figure 1:**
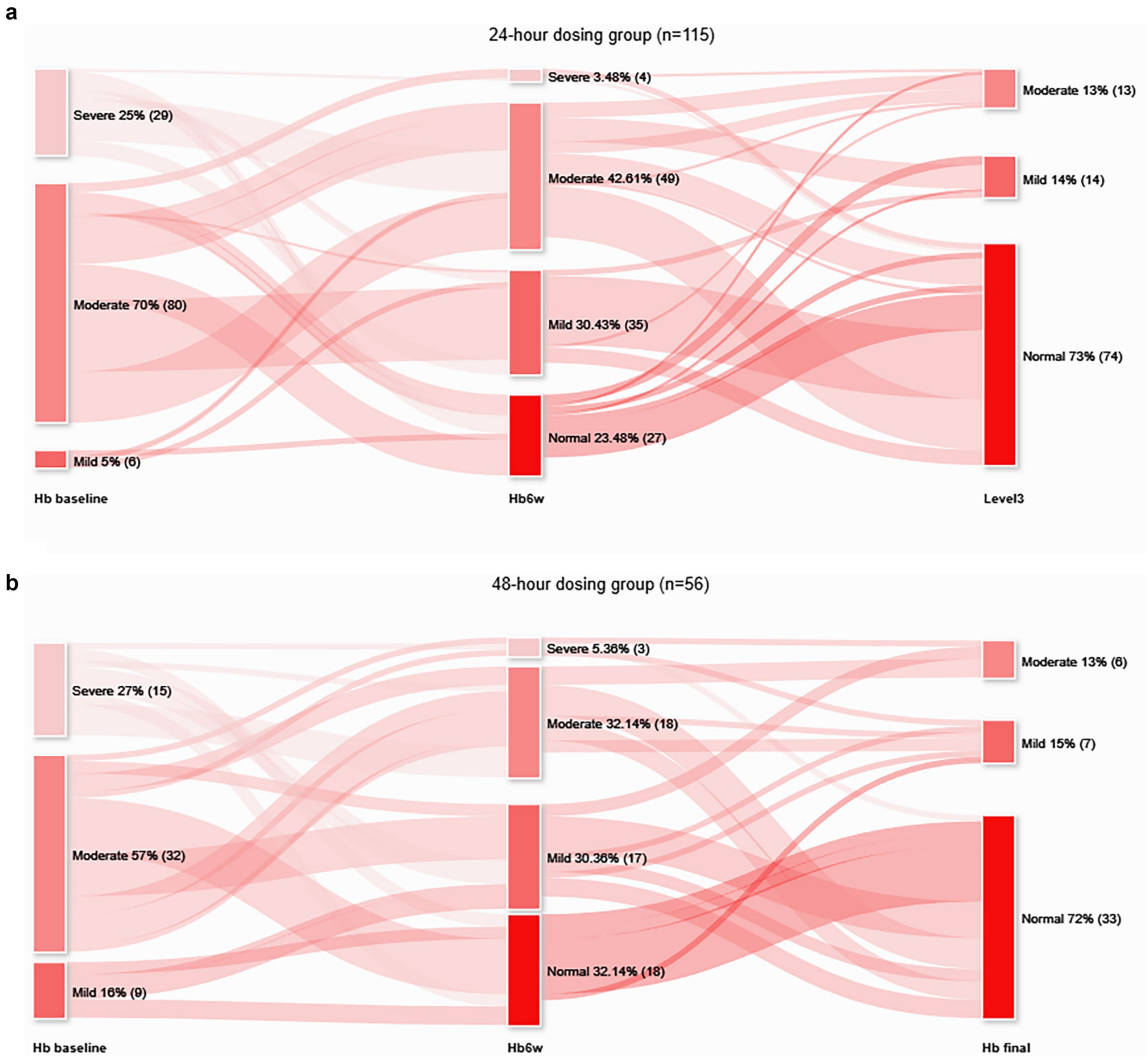
Sankey diagrams showing hemoglobin severity transitions from baseline through 6 weeks to the final 3-month assessment in **(a)** the 24-h dosing group and **(b)** the 48-h dosing group.

Final hemoglobin measurements at 3 months were available for 147 patients, including 101 in the daily dosing group and 46 in the alternate-day group. Mean final hemoglobin was 12.79 ± 1.68 g/dL in the daily dosing group and 12.68 ± 1.62 g/dL in the alternate-day group, without a statistically significant between-group difference. Hemoglobin normalization was observed in 76 patients (75.2%) in the daily dosing group and 33 patients (71.7%) in the alternate-day group. Baseline and follow-up hematologic outcomes according to oral iron dosing schedule are summarized in Table 3. No imputation was performed for missing follow-up values. These findings are represented in Figures 1a,b

**Table 3:** Hematologic outcomes according to oral iron dosing schedule.

Variable	Daily dosing *n* = 115	Alternate-day dosing *n* = 56	*p*-value
Baseline hemoglobin			
Hemoglobin, g/dL	8.84 ± 1.38	9.38 ± 1.54	0.026
Anemia severity at baseline			
Severe anemia	29 (25.2)	15 (26.8)	0.050
Moderate anemia	80 (69.6)	32 (57.1)	
Mild anemia	6 (5.2)	9 (16.1)	
Six-week evaluation			
Hemoglobin at 6 weeks, g/dL	10.91 ± 1.76	11.26 ± 1.86	0.255
Anemia severity at 6 weeks			
Severe anemia	4 (3.5)	3 (5.4)	0.484
Moderate anemia	49 (42.6)	18 (32.1)	
Mild anemia	35 (30.4)	17 (30.4)	
Normal hemoglobin	27 (23.5)	18 (32.1)	
Three-month evaluation			
Final hemoglobin, g/dL	12.79 ± 1.68	12.68 ± 1.62	0.692
Anemia severity at 3 months			
Moderate anemia	13 (12.9)	6 (13.0)	0.975
Mild anemia	14 (13.9)	7 (15.2)	
Normal hemoglobin	74 (73.3)	33 (71.7)	
Hemoglobin normalization ≥12 g/dL	76 (75.2)	33 (71.7)	0.687

Data are presented as mean ± standard deviation or *n* (%), as appropriate. Percentages for baseline and 6-week outcomes were calculated using the total number of patients in each dosing group. Percentages for 3-month outcomes were calculated among patients with available final hemoglobin measurements. *p*-values for anemia severity correspond to the overall comparison of the distribution of severity categories between groups, not to each individual category. No imputation was performed for missing final hemoglobin values.

A sensitivity analysis compared patients with available final hemoglobin measurements at 3 months (*n* = 147) with those without final hemoglobin data (*n* = 24). There were no statistically significant differences between completers and non-completers in age (39.42 ± 8.82 vs. 38.29 ± 11.49 years; *p* = 0.649), baseline hemoglobin (9.00 ± 1.42 vs. 9.15 ± 1.65 g/dL; *p* = 0.677), alternate-day dosing assignment (31.3% vs. 41.7%; *p* = 0.352), comorbidity status (32.7% vs. 25.0%; *p* = 0.636), baseline ferritin availability (61.2% vs. 54.2%; *p* = 0.510), or baseline ferritin values among those with available measurements (median, 8.55 vs. 8.24 ng/mL). However, baseline anemia severity distribution differed between groups (*p* = 0.028), with a higher proportion of severe anemia among non-completers. Therefore, available-case analysis was used for final hemoglobin outcomes because no follow-up hemoglobin data were available for these patients, and potential attrition bias cannot be excluded.

### 3.3 Safety and treatment-related adverse events

High adherence was observed in 97 patients (84.3%) receiving daily dosing and in 44 patients (78.6%) receiving alternate-day dosing, without a statistically significant between-group difference.

Treatment discontinuation due to adverse events was uncommon and occurred in 9 patients (7.8%) in the daily dosing group and 6 patients (10.7%) in the alternate-day group. Escalation to intravenous iron was required in 16 patients (13.9%) receiving daily dosing and 15 patients (26.8%) receiving alternate-day dosing; this difference did not reach conventional statistical significance using Fisher’s exact test (OR, 2.26; 95% CI, 1.02–5.00; *p* = 0.056). Adherence, treatment discontinuation, and intravenous iron escalation according to dosing schedule are summarized in Table 4.

**Table 4:** Adherence, treatment discontinuation, and intravenous iron escalation according to oral iron dosing schedule.

Variable	24-h dosing *n* = 115	48-h dosing *n* = 56	OR (95% CI)	*p*-value
Adherence ≥80% Adherence <80%	97 (84.3) 18 (15.7)	44 (78.6) 12 (21.4)	1.47 (0.65–3.31)	0.394
Treatment discontinuation due to adverse events	9 (7.8)	6 (10.7)	0.71 (0.24–2.10)	0.570
Intravenous iron escalation	16 (13.9)	15 (26.8)	2.26 (1.02–5.00)	0.056

Data are presented as mean ± standard deviation or *n* (%), as appropriate. Percentages were calculated using the corresponding group denominator.

### 3.4 Factors influencing hematologic recovery (≥12 g/dL)

Factors potentially associated with hemoglobin normalization (Hb ≥ 12 g/dL) were explored, including treatment schedule, therapeutic adherence, comorbidity burden, and endocrinologic disorders. Normalization rates were similar between the daily and alternate-day regimens (75.2% vs. 71.7%; OR, 1.20; 95% CI, 0.55–2.63; *p* = 0.687). Adherent patients showed a similar normalization rate compared with non-adherent patients (74.4% vs. 72.0%; OR, 1.13; 95% CI, 0.43–2.96; *p* = 0.805). Hemoglobin normalization also did not differ significantly according to comorbidity status (77.1% with ≥1 comorbidity vs. 72.7% without comorbidities; OR, 1.26; 95% CI, 0.56–2.82; *p* = 0.689) or endocrinologic disorders (72.7% with endocrine or metabolic comorbidities vs. 74.4% without; OR, 0.92; 95% CI, 0.33–2.55; *p* = 1.000). Overall, none of the evaluated factors was significantly associated with hemoglobin normalization.

### 3.5 Exploratory adjusted analyses

In exploratory adjusted analyses including dosing schedule, baseline hemoglobin concentration, age, and comorbidity status, alternate-day dosing was not significantly associated with final hemoglobin concentration at 3 months (adjusted *β*, −0.20 g/dL; 95% CI, −0.80 to 0.39; *p* = 0.497) or hemoglobin normalization (adjusted OR, 0.76; 95% CI, 0.33–1.73; *p* = 0.512). In contrast, alternate-day dosing was associated with higher odds of intravenous iron escalation after adjustment (adjusted OR, 2.29; 95% CI, 1.02–5.16; *p* = 0.045). However, this finding should be interpreted cautiously because intravenous iron escalation was a secondary, non-standardized real-world clinical outcome.

## 4 Discussion

Iron deficiency anemia is particularly relevant in women of reproductive age, in whom chronic gynecologic blood loss represents one of the most frequent clinical contexts. In the present cohort, iron deficiency anemia was predominantly associated with abnormal uterine bleeding, and leiomyomas were the most frequent etiology according to the PALM–COEIN classification. This finding is consistent with previous evidence identifying heavy menstrual bleeding as a major contributor to iron deficiency and iron deficiency anemia in women, with important effects on quality of life, physical functioning, work performance, and productivity (11, 12). Therefore, the hematologic response observed in this study should be interpreted within a population in which ongoing or recurrent blood loss may influence treatment response independently of the oral iron dosing schedule (13, 14).

Oral iron supplementation remains the standard first-line treatment for most patients with iron deficiency anemia because of its availability, low cost, and overall safety profile (15). However, its effectiveness in routine practice may be influenced by elemental iron dose, formulation, gastrointestinal tolerance, interactions with food or medications, adherence, and the need for treatment intensification in selected patients (16–20). In this real-world cohort, both daily and alternate-day oral iron regimens were associated with hematologic improvement during follow-up. Mean hemoglobin increased in both groups, and no statistically significant between-group differences were observed in hemoglobin concentration at 6 weeks, final hemoglobin concentration at 3 months, or hemoglobin normalization.

These findings should be interpreted cautiously. Treatment allocation was not randomized and was determined by physician judgment during routine clinical practice. Patients receiving daily dosing had lower baseline hemoglobin values and a borderline difference in baseline anemia severity distribution compared with those receiving alternate-day dosing. This suggests that physicians may have preferentially prescribed daily therapy to patients perceived as having more severe anemia, while alternate-day dosing may have been selected for patients considered clinically less severe, more prone to intolerance, or more suitable for a less intensive regimen. Therefore, the apparent similarity in hematologic outcomes should not be interpreted as evidence of therapeutic equivalence or non-inferiority between daily and alternate-day dosing. Rather, these findings indicate that both regimens were associated with hemoglobin improvement in routine clinical practice, while residual confounding and selection bias remain possible.

The rationale for alternate-day dosing is largely based on prior physiological evidence involving hepcidin-mediated regulation of iron absorption. Stoffel et al. (21) reported that alternate-day iron administration may improve fractional iron absorption compared with consecutive-day dosing in iron-depleted women. Other studies have described hepcidin as a central regulator of iron metabolism and a potential mediator of reduced absorption after repeated or higher-dose oral iron exposure (22). In the present study, the alternate-day regimen resulted in lower cumulative oral iron exposure than daily dosing. However, hepcidin concentrations and fractional iron absorption were not measured; therefore, these mechanisms are cited only as biological background for alternate-day dosing and should not be interpreted as mechanisms demonstrated by our data.

Our findings should also be interpreted in relation to previous studies comparing oral iron schedules. Uçan et al. evaluated daily and alternate-day oral iron regimens and reported that alternate-day administration with 120 mg elemental iron was associated with a greater median hemoglobin increase, although gastrointestinal adverse events were also more frequent in that regimen (23). In contrast, Caştur et al. (24) compared different oral iron dosing schedules in premenopausal women and found improvement across all groups, with variability in hematologic response and tolerability according to regimen. Similar uncertainty regarding the clinical superiority of alternate-day regimens has also been reported in other populations, including pregnant women (25). Together with the present study, these findings suggest that the effect of dosing interval may depend on elemental iron dose, baseline anemia severity, treatment duration, patient selection, adherence assessment, and the presence of ongoing blood loss.

The expected tolerability advantage of alternate-day dosing was not clearly observed in this cohort. High adherence was frequent in both groups, and treatment discontinuation due to adverse events was uncommon and similar between dosing schedules. This may reflect selection bias inherent to non-randomized real-world practice. Physicians may have preferentially prescribed alternate-day therapy to patients perceived to be at higher risk of intolerance, including those with previous gastrointestinal symptoms, prior poor tolerance to oral iron, concern for poor adherence, or patient preference for a less frequent regimen. Such selection could have increased the baseline risk of adverse events in the alternate-day group and reduced the likelihood of observing a clear tolerability difference between schedules.

Escalation to intravenous iron was numerically more frequent among patients receiving alternate-day dosing than among those receiving daily dosing. However, this finding should be interpreted cautiously because it was a secondary outcome, the statistical evidence was borderline, and escalation decisions were made during routine clinical care rather than according to a standardized institutional protocol. Therefore, intravenous iron use should not be interpreted as a direct or definitive measure of oral iron failure. Instead, it reflects real-world treatment intensification based on clinical judgment, including inadequate hematologic response, persistent symptoms, ongoing abnormal uterine bleeding, intolerance to oral therapy, or need for faster correction. This observation reinforces the importance of close monitoring and individualized treatment adjustment, consistent with current approaches that emphasize tailoring iron therapy according to severity, response, tolerability, and treatment goals (17, 26).

In exploratory analyses, hemoglobin normalization was not significantly associated with dosing schedule, adherence, comorbidity burden, or endocrine/metabolic comorbidities. These results should be considered hypothesis-generating. The lack of statistically significant associations may reflect limited statistical power, the high overall adherence observed in the cohort, heterogeneity in bleeding severity, and the influence of unmeasured factors such as baseline iron deficit, dietary intake, gastrointestinal absorption, and timing of gynecologic treatment. Therefore, the absence of significant predictors should not be interpreted as evidence that these factors are clinically irrelevant.

The main strength of this study is its pragmatic, real-world design in a clinically relevant population of women with iron deficiency anemia, predominantly associated with abnormal uterine bleeding. The study also incorporated outcomes relevant to routine practice, including adherence, adverse-event-related discontinuation, follow-up hemoglobin response, anemia severity categories, and escalation to intravenous iron.

This study has several limitations. First, the observational and non-randomized design introduces selection bias and residual confounding. Treatment allocation was based on physician judgment and patient-level factors rather than random assignment, and baseline hemoglobin was lower in the daily dosing group. As a result, the study cannot establish equivalence, non-inferiority, or causal superiority of either dosing schedule. Second, no formal *a priori* sample size calculation or power analysis was performed, as the study included consecutive eligible patients treated during the study period. Therefore, the study should be considered exploratory and hypothesis-generating. The available sample may have been underpowered to detect clinically meaningful differences between groups, particularly for final hemoglobin concentration and intravenous iron escalation. Accordingly, non-significant *p*-values should not be interpreted as evidence of equivalence or absence of clinically relevant differences. Third, ferritin and transferrin saturation were not systematically available for all participants. Baseline ferritin was available in 103 patients, and some patients had ferritin values ≥30 ng/mL; in these cases, iron deficiency was confirmed using complementary iron studies and clinical assessment as part of routine practice. Nevertheless, diagnostic heterogeneity cannot be fully excluded, and the findings should be interpreted primarily as reflecting hemoglobin recovery rather than complete biochemical characterization of iron deficiency or iron-store repletion. Fourth, final hemoglobin values were missing in 24 patients and were handled using available-case analysis because no follow-up hemoglobin data were available for these individuals. A sensitivity analysis showed no significant differences between patients with and without final hemoglobin measurements in age, baseline hemoglobin, treatment group, comorbidity status, ferritin availability, or baseline ferritin values. However, non-completers had a higher proportion of severe anemia at baseline; therefore, attrition bias cannot be fully excluded. Fifth, criteria for intravenous iron escalation were based on clinical judgment rather than a standardized protocol, which may have introduced variability in treatment intensification decisions. Finally, adherence was assessed using pill counts and patient-reported questionnaires, which may be affected by recall and social desirability bias; the ≥80% adherence threshold was used as a pragmatic cut-off and was not evaluated through alternative adherence thresholds.

In conclusion, daily and alternate-day oral iron regimens were both associated with hematologic improvement during follow-up in this real-world cohort of women with iron deficiency anemia. However, because treatment allocation was non-randomized, baseline differences were present, and the study was not designed to establish equivalence or non-inferiority, the results should be interpreted cautiously. Alternate-day dosing did not show a clear tolerability advantage and was associated with numerically higher intravenous iron escalation; however, this secondary outcome should be interpreted cautiously because escalation decisions were based on clinical judgment without standardized criteria. Oral iron strategies should be individualized according to baseline anemia severity, tolerability, adherence, ongoing blood loss, and treatment goals. Prospective randomized studies with adequate sample size, standardized escalation criteria, systematic iron-parameter assessment, and longer follow-up are needed to identify which patients are most likely to benefit from daily or alternate-day dosing strategies.

## Data Availability

Due to privacy and confidentiality restrictions, the data presented in this study are available from the corresponding author upon reasonable request. The data are not publicly available due to the confidentiality requirements established by the Research and Ethics Committees of the Hospital General de México ‘Dr. Eduardo Liceaga.
